# The Papanicolaou Smear Reimagined: A Narrative Review of Cervicovaginal Cytology and Molecular Biospecimens for Ovarian Cancer Detection

**DOI:** 10.3390/medicina62050873

**Published:** 2026-05-02

**Authors:** Andrej Cokan, Leyla Al Mahdawi, Manuela Ludovisi, Maja Pakiž, Jure Knez, Andraž Dovnik

**Affiliations:** 1 Department of Gynaecological and Breast Oncology, University Medical Centre Maribor, 2000 Maribor, Slovenia; leylaalmahdawi@ukc-mb.si (L.A.M.); maja.pakiz@ukc-mb.si (M.P.); jure.knez@ukc-mb.si (J.K.); andraz.dovnik@ukc-mb.si (A.D.); 2 Faculty of Medicine, University of Maribor, 2000 Maribor, Slovenia; 3 Department of Life Health and Environmental Sciences, University of L’Aquila, 67100 L’Aquila, Italy; mludovisi@gmail.com

**Keywords:** cervicovaginal cytology, DNA methylation, molecular biomarkers, liquid biopsy, early detection

## Abstract

The Papanicolaou (Pap) smear, a cornerstone of cervical cancer prevention, has emerged as a compelling, though unconventional, biospecimen for the detection of ovarian cancer (OC). This structured narrative review synthesizes the evolving evidence on the utility of cervicovaginal cytology and molecular analysis of Pap test material for OC detection. While conventional cytology provides a proof of concept, its sensitivity is low, ranging from incidental detection of OC in 0.004% of routine screens to 19.3% in patients with known OC. Specific cytologic findings, however, carry significant predictive value: atypical glandular cells (AGC) confer a two-fold increased OC risk, and psammoma bodies (PB) are strongly associated with serous malignancies. Driven by the sensitivity limitations of morphology, the field has undergone a paradigm shift towards molecular detection. Foundational studies confirmed tumor-derived DNA, including hallmark TP53 mutations, is detectable in Pap samples years before diagnosis, though sensitivity is constrained by low DNA abundance and confounded by background clonal mutations. To overcome this, strategies have expanded to target broader genomic signatures, such as somatic copy number alterations (EVA test: 75% sensitivity, 96% specificity), and multi-gene mutation panels (PapSEEK: 33–45% sensitivity). The most promising advances lie in multi-omic approaches, particularly DNA methylation biomarkers, which have demonstrated sensitivities up to 81% with high specificity. Collectively, this evidence argues against repurposing the Pap test as a standalone OC screen but supports its strategic integration into a risk-stratified clinical algorithm. We propose a “reflex-to-molecular” model where high-risk cytology (e.g., AGC, PB) automatically triggers advanced molecular testing on the same sample. This model efficiently leverages existing infrastructure to triage high-risk women for definitive diagnostics. Prospective validation of this integrated approach is the essential next step toward transforming this test into a sentinel for malignancies of the upper female reproductive tract.

## 1. Introduction

Ovarian cancer (OC) remains one of the most lethal gynecologic malignancies, with an estimated 324,398 new cases and 206,839 deaths worldwide in 2022, reflecting a persistently high mortality-to-incidence ratio [1]. Notably, both incidence and mortality show marked geographic and socioeconomic disparities, with higher mortality rates observed in low- and middle-income countries, largely due to limited access to specialized care, delayed diagnosis, and reduced availability of diagnostic pathways for early evaluation. These disparities are relevant when considering the global applicability of emerging molecular screening strategies. A primary driver of this poor prognosis is the advanced stage at diagnosis for the predominant and most aggressive subtype, high-grade serous carcinoma (HGSOC). Most serous carcinomas are diagnosed at stage III (51%) or IV (29%), for which the 5-year cause-specific survival is only 42% and 26%, respectively [2].

Despite its clinical impact, no population-wide screening program has proven successful in reducing mortality. Large randomized trials, such as the UK Collaborative Trial of Ovarian Cancer Screening (UKCTOCS), which evaluated multimodal screening with serum CA-125 and transvaginal ultrasound, failed to demonstrate a significant mortality reduction [3]. For the general population, opportunistic salpingectomy has emerged as a preventive strategy based on the fallopian tube origin of most HGSOC, with population data suggesting a marked reduction in serous OC incidence after the procedure [4]. However, even effective preventive strategies cannot address all at-risk populations, including those who are not surgical candidates, leaving a persistent gap in early detection. This demonstrates that primary prevention is a powerful but incomplete strategy in the absence of effective screening.

In this challenging landscape, the Papanicolaou (Pap) smear presents a uniquely compelling, though unconventional, avenue for investigation. As a routine, minimally invasive, and widely accepted cervical cancer screening test, its potential retooling to detect molecular or cellular signatures of OC could reshape early detection paradigms. The biological rationale is grounded in the concept that exfoliated cells or biomolecules from the fallopian tubes, endometrium, and ovaries can traverse the genital tract and be captured in cervicovaginal samples, offering a “liquid biopsy” of the upper female reproductive tract [5].

This narrative review synthesizes the current evidence on the utility of the Pap smear and related cervicovaginal biospecimens in the context of OC. We examine the diagnostic and prognostic value of traditional cytomorphologic findings, such as atypical glandular cells (AGC) and psammoma bodies (PB). Furthermore, we explore the evolving frontier of molecular analysis performed on Pap test material, including the detection of somatic mutations (e.g., TP53), epigenetic alterations (DNA methylation), and proteomic profiles. By integrating this body of evidence, we aim to delineate the current capabilities, highlight persistent challenges (particularly regarding sensitivity and specificity) and discuss future research directions necessary to evaluate whether this simple, accessible test can contribute to reducing the mortality of this deadly disease.

## 2. Materials and Methods

We conducted a structured narrative review to explore the diagnostic and prognostic utility of the Pap smear and related cervicovaginal cytology in detecting OC. The objective was to provide a comprehensive and clinically relevant synthesis of the available evidence, identify emerging molecular approaches, and highlight key gaps to guide future research. While a structured search strategy was applied to enhance transparency and reproducibility, this review was not designed to meet the methodological requirements of a formal systematic review (e.g., protocol registration or formal risk-of-bias assessment), and the synthesis reflects a narrative interpretation of the literature. As this was a narrative review, formal ethical approval was not required and no human subject data were collected.

### 2.1. Literature Search Strategy and Study Selection

A structured literature search was performed using the PubMed/MEDLINE database. The search strategy combined Medical Subject Headings (MeSH) and free-text terms, including: (“ovarian neoplasms”[MeSH Terms] OR “ovarian cancer”) AND (“Papanicolaou Test”[MeSH Terms] OR “pap smear” OR “Papanicolaou smear” OR “liquid based cytology”). Additional searches were conducted in the Wiley Online Library and the Cochrane Library using relevant keyword combinations.

The search included studies published up to November 2025. Reference lists of relevant articles were also screened to identify additional studies not captured in the initial search. Publications were limited to the English language. Exclusion criteria included review articles, ongoing studies without published results, conference abstracts, oral presentations, and articles without accessible full text. All eligibility criteria, including exclusion of review articles and other non-original study types, were applied during the screening phase rather than as database-level filters.

All identified records were imported into an Excel database for screening. After removal of duplicates, two reviewers (A.C. and A.Ž.) independently screened titles and abstracts for relevance. Potentially eligible studies were retrieved in full text and assessed against the inclusion criteria. Discrepancies were resolved through discussion, with involvement of additional reviewers (M.P. and J.K.) when necessary to reach consensus. In addition, four supplementary studies providing general epidemiological and clinical background context on OC were included to support interpretation of the findings; these were not derived from the search process.

To enhance transparency of the study selection process, a flow diagram is provided (Supplement Figure S1). However, given the narrative nature of this review, this diagram is intended for illustrative purposes and does not imply full adherence to PRISMA guidelines.

### 2.2. Limitations of the Methodology

This review has several methodological limitations. As a narrative review, it lacks the formal methodological rigor of a systematic review, including protocol registration and structured risk-of-bias assessment. The search strategy, while structured, was limited to selected databases (PubMed/MEDLINE, Wiley, and Cochrane), and the exclusion of EMBASE and Scopus may have resulted in incomplete capture of relevant literature. Additionally, restriction to English-language publications introduces potential language bias. The included evidence is heterogeneous, comprising case reports, small observational studies, and predominantly retrospective analyses, which may introduce selection bias and limit the generalizability of findings.

## 3. Results

### 3.1. Cytological Evidence and Diagnostic Utility of Pap Smear for Extrauterine Malignancies

#### 3.1.1. General Detection of Extrauterine Malignancies

The potential for extrauterine malignancies to be incidentally detected via cervical or vaginal cytology was first highlighted in a 1949 report of ovarian adenocarcinoma cells in a vaginal smear [6]. Subsequent case reports and retrospective studies have confirmed that the detection of extrauterine malignancies in cervical Pap smears is extremely rare, with reported rates ranging from 0.004% to 0.8% [7,8].

Gupta et al., in a study of approximately 900,000 evaluated Pap smears, identified 33 cases (0.004%) of abnormal Pap smears originating in histologically proven extrauterine carcinomas [7]. The most common primary sites were the ovary (42%), gastrointestinal tract (18%), and breast (18%). A larger contemporary series by Tandon et al., evaluating 11,674 Pap smears, found 104 (0.8%) with extrauterine malignancy. In this series, a definitive diagnosis of extrauterine malignancy was rendered in 47.1% of cases, while 30.9% were reported as positive for malignancy without specifying origin, and 22.0% were reported as AGC only [8].

In dedicated studies of patients with known OC, Takashina et al. cytologically examined 114 patients who received preoperative evaluation [9]. The overall positive cytologic rate was 26.3% (30/114), with 19.3% (22/114) positive in cervicovaginal smears specifically. The presence of ascites, rather than its volume, significantly increased the cytologic detection rate by 2.1 times, suggesting OC cells may reach the cervix via trans-tubal dissemination, particularly in the presence of ascites.

Regarding histology, serous cystadenocarcinoma was the dominant type, present in 33.3% of cases in the Takashina study and 68% in the Tandon study [8,9]. The cytomorphology of metastatic ovarian tumors in Pap smears, as described by Tandon et al., typically includes papillary fragments with scalloped edges, high nucleus-to-cytoplasm ratios, coarse chromatin, and prominent nucleoli, often displaying greater nuclear atypia compared to primary cervical adenocarcinomas [8].

To improve diagnostic accuracy, ancillary techniques such as immunohistochemistry (IHC) have been applied. Baars et al. demonstrated that CK7 immunostaining on Pap-stained cytology can support the identification of ovarian carcinoma cells [10]; however, CK7 alone lacks specificity and cannot reliably distinguish ovarian from other CK7-positive adenocarcinomas (e.g., pancreatic or biliary). Therefore, a broader immunohistochemical panel (CK7+/CK20−/PAX8+), is preferred to more accurately determine tumor origin.

In summary, while conventional cytology provides proof-of-concept for the transtubal shedding and retrieval of OC cells, its detection rate is low, ranging from 0.004% in routine screening [7] to 19.3% in cervicovaginal smears from patients with known OC [9]. This established sensitivity limitation of morphology-based detection, coupled with the interpretive challenge of atypical cells, provides a compelling rationale for the development of more sensitive and objective molecular diagnostic strategies.

#### 3.1.2. Predictive Value of AGC

Beyond incidental findings, specific cytologic interpretations carry significant predictive value. The Pap test’s utility in detecting OC is quantified through large cohort studies of women with AGC, a rare cytologic finding with an incidence ranging from 0.09% to 0.41% in screened populations [11,12,13,14,15]. This finding is a marker for underlying malignancy, conferring a two-fold increased risk of OC (SIR 2.04, 95% CI 1.05–3.56) compared with the age-appropriate general female population derived from the Taiwanese national cancer registry, based on women undergoing routine cervical screening within the national screening programme between 1995 and 2004 with cancer incidence follow-up through 2006 [16]; however, the lower bound of the confidence interval is close to 1.0, suggesting borderline statistical significance. The proportion of AGC patients diagnosed with OC varies across studies, from 0.6% to 2.5% [11,12,13,17]. In large series, ovarian or tubal carcinomas constituted 2.1% of all AGC-associated malignancies [15]. Risk is powerfully stratified by cytologic subclass, with “AGC-favor neoplastic” (AGC-FN) carrying the highest burden; Kim et al. reported OC in 7.1% of AGC-FN patients versus 3.6% in the “AGC-not otherwise specified” (AGC-NOS) group [18]. Furthermore, ovarian malignancies are associated more with HPV-negative AGC results, as demonstrated by Zhao et al., where the sole OC in their series was HPV-negative [19]. When AGC is linked to OC, it often indicates advanced disease; Xu et al. detailed the cytomorphology of HGSOC in Pap smears and noted 75% of such patients presented with Stage IIIB-IV disease [20]. Consequently, when cervical and endometrial evaluations are negative in an AGC patient, underlying ovarian pathology must be definitively ruled out [14].

In summary, while AGC is an uncommon Pap result, it serves as an epidemiologic and diagnostic indicator, identifying a subset of women with a measurably elevated risk of OC, particularly those with AGC-FN or HPV-negative results, necessitating a diagnostic investigation of the adnexa.

#### 3.1.3. Association of PB

The incidental detection of PB in a routine Pap smear is an exceptionally rare event, but one with potential clinical significance due to its strong association with serous carcinomas of the ovary, fallopian tube, peritoneum, and endometrium. The prevalence of PB is extraordinarily low, ranging from 0.0006% (10/1,497,540) in a 31-year review [21] to 0.05% (18/34,816) in a 4-year cohort [22]. When identified, the predictive value for malignancy is heavily influenced by menopausal status and concurrent cytologic findings. Zreik et al. reported a highly ominous association in postmenopausal women, where 7 of 8 (87.5%) with PB had a gynecologic malignancy, including serous OC, fallopian tube carcinoma, and uterine serous or clear-cell carcinomas; however, this finding is based on a very small sample size and should therefore be interpreted with caution. The presence of concurrent AGC or malignant cells is a powerful predictor, present in all malignant cases in Zreik’s series [22] and in 50% (2/4) of the borderline/malignant cases reported by Ersoy et al. [21]. However, PB alone could also be important, especially in patients with a relevant family history of cancer or genetic mutations (e.g., BRCA).

In addition to ovarian and tubal cancers, primary peritoneal serous psammocarcinoma (a rare variant characterized by massive psammoma body formation) has been diagnosed following the detection of PB and neoplastic cells on Pap smear, as detailed in the case report by Riboni et al. [23].

The collective evidence mandates that the finding of PB, whether isolated or accompanied by glandular abnormalities, should trigger a diagnostic evaluation of the entire female genital tract and peritoneum, as it can be the first and only cytologic clue to an otherwise occult serous neoplasm.

### 3.2. Foundational Molecular Evidence: The Shift to DNA-Based Detection

The limitations of conventional cytology catalyzed a paradigm shift, moving from microscopic identification of intact cells to the molecular detection of tumor-derived genetic material within Pap test samples. The seminal study by Kinde et al. provided the foundational proof-of-concept for this approach [24].

This groundbreaking work employed a tumor-informed, ultra-sensitive sequencing strategy, Safe-Sequencing System (Safe-SeqS), capable of detecting a single mutant allele among thousands to millions of wild-type templates. The researchers first identified patient-specific somatic mutations (most frequently in TP53, ARID1A, PIK3CA, and KRAS) via sequencing of tumor tissue from 22 OC patients. They then analyzed DNA from matched liquid-based Pap smear specimens.

The results were transformative: tumor-specific mutations identified in the primary tumor were also detected in Pap smear DNA. Detection rates, however, differed markedly by tumor type. Using a sensitive massively parallel sequencing method, mutations were identified in 100% of endometrial cancers (24/24) but in only 41% of OCs (9/22), highlighting the greater biological challenge of detecting OC through cervicovaginal samples. Furthermore, the mutant allele fraction in OC Pap samples was significantly lower (median 0.49%) than in EC, indicating a much sparser and more stochastic shedding of tumor DNA into the cervical canal, a finding consistent with the greater anatomical distance from the adnexa.

The Kinde et al. study demonstrated two critical principles: (1) tumor-derived DNA from ovarian carcinomas is present and detectable in routine Pap smears, and (2) the low fractional abundance of this DNA presents a primary obstacle for clinical detection. This work established the technical blueprint and defined the core sensitivity challenge that all subsequent molecular strategies (from targeted TP53 assays to multi-gene panels and copy-number analyses) have sought to overcome.

From a methodological perspective, this progression also reflects increasing analytical sensitivity, from early error-corrected sequencing approaches such as Safe-SeqS, to high-depth duplex sequencing and later targeted amplification methods such as droplet digital PCR (ddPCR), each offering improved limits of detection for low-abundance tumor DNA in cervicovaginal samples.

### 3.3. Tumor Suppressor Protein TP53

The feasibility of detecting tumor-derived TP53 mutations in cervicovaginal samples for the early diagnosis of HGSOC is supported by longitudinal evidence. In a retrospective cohort study of 17 HGSOC patients, Paracchini et al. used ddPCR and found that clonal pathogenic TP53 variants identical to the primary tumor were detectable in archival, cytologically negative Pap test samples collected up to 6 years prior to diagnosis [25]. The variant was detected in 11 of 17 patients (64.7%), including in longitudinal samples from two patients, providing critical experimental evidence for a multi-year window for potential molecular early detection. Further exploring the limits of technical sensitivity, Arildsen et al. conducted a retrospective analysis of liquid-based Pap samples from 15 HGSOC patients using an ultra-sensitive ddPCR method (IBSAFE) capable of detecting mutant alleles at frequencies as low as 0.0068% with minimal DNA input [26]. This method identified the tumor-matched mutation in diagnostic samples from 6 of 8 patients (75%) with a known somatic TP53 alteration. Notably, in one patient, the IBSAFE assay detected the mutation in an archival sample collected 20 months prior to diagnosis, though not in samples taken earlier, hinting at a potential narrower window for reliable pre-symptomatic detection.

It should be noted that these foundational studies are generally retrospective and involve small cohorts with heterogeneous pre-analytical conditions (including variation in sample type, storage duration, and DNA extraction protocols), which may introduce variability and limit comparability across studies; formal risk-of-bias assessment frameworks were not applied across all included studies, which should be considered when interpreting the strength of the evidence.

**Figure 1:**
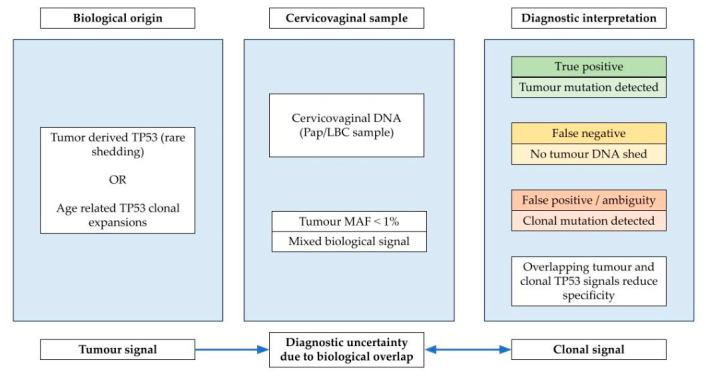
Clonal TP53 mutations in cervicovaginal samples arise from both tumor-derived shedding and age-related clonal expansions, creating diagnostic ambiguity that reduces specificity for mutation-based ovarian cancer detection.

However, the overarching sensitivity for detecting the singular tumor-driver mutation in cervicovaginal samples remains a significant challenge, influenced by anatomical distance and stochastic shedding of tumor material. Krimmel-Morrison et al., in a prospective analysis using ultra-deep duplex sequencing (~3000× depth) on Pap test DNA from 30 women (9 with HGSOC), identified the matching tumor mutation in only 3 of 8 (37.5%) cancer patients with intact tubes, aligning with prior reports. This indicates that the limitation is often biological (absence of tumor DNA in the sample) rather than purely technical. A critical complication for the specificity of mutation-based diagnostics is the discovery of widespread, low-frequency somatic TP53 mutations in non-cancerous tissues due to age-related clonal evolution. Krimmel-Morrison et al. first detailed this in Pap tests, finding abundant, cancer-like TP53 mutations (enriched in hotspots and predicted pathogenic) in samples from women both with and without OC (Figure 1). This finding challenges the simple binary interpretation of a TP53 mutation’s presence [27].

A subsequent, more comprehensive retrospective case–control study by Ghezelayagh et al., analyzing 70 individuals with ultra-deep duplex sequencing of matched blood and liquid-based cytology (LBC) samples, confirmed the low sensitivity (30.4%) for the tumor mutation in LBC but extensively mapped this background somatic landscape [28]. They found pathogenic TP53 mutations in nearly all LBC and blood samples, with only 5.4% of LBC mutations shared with blood, indicating distinct clonal expansions in the gynecological tract. Crucially, their analysis revealed that BRCA1/2 pathogenic variant (BRCApv) carriers who developed HGSOC had a significantly higher burden of pathogenic TP53 clones in their LBC samples compared to BRCApv carriers without cancer. This association persisted after removing tumor-specific or blood-concordant mutations, suggesting that an elevated burden of TP53-mutant clones in the reproductive tract may reflect a field defect or increased cancer risk in genetically predisposed individuals.

These findings highlight a fundamental limitation of mutation-based early detection strategies: the challenge of distinguishing true tumor-derived mutations from background clonal expansions. In cervicovaginal samples, tumor-derived TP53 mutations are typically present at very low mutant allele fractions (MAFs), as demonstrated in sequencing-based studies (e.g., median ~0.49% in OC Pap samples), reflecting the sparse and intermittent shedding of tumor DNA from the adnexa. In parallel, age-related clonal processes (including clonal hematopoiesis of indeterminate potential (CHIP) and localized epithelial clonal evolution) can generate detectable TP53 mutations in non-malignant tissues, creating a biologically complex background signal that overlaps with true tumor-derived alterations and limits specificity.

One potential strategy to mitigate this challenge is the incorporation of paired blood sequencing, as demonstrated by Ghezelayagh et al., which enables identification and exclusion of mutations arising from hematopoietic clones. However, their data also indicate that most TP53 mutations detected in cervicovaginal samples are not blood-derived, suggesting that local clonal expansions within the gynecologic tract represent an additional and unresolved source of background noise. Notably, this mutational landscape appears to differ by risk group: BRCA1/2 pathogenic variant carriers, particularly those who develop HGSOC, exhibit a higher burden of pathogenic TP53 clones, potentially reflecting a field effect or increased genomic instability in susceptible tissues.

Collectively, these observations underscore that clinical implementation is constrained not only by sensitivity but also, critically, by specificity, which is compromised by background clonal mutations in cervicovaginal samples. Future diagnostic approaches will therefore likely require integrative strategies that move beyond single-mutation detection and combine mutation-based analysis with orthogonal genomic and epigenetic biomarkers, such as DNA methylation patterns and somatic copy number alterations. Such frameworks will also need to incorporate quantitative parameters, including allele fraction, mutation burden, and mutational patterns, to reliably distinguish malignant from non-malignant clonal signals.

In this context, TP53 remains the central molecular hallmark of HGSOC and a compelling target for early detection. While proof-of-concept studies demonstrate that tumor-derived TP53 mutations can be detected in Pap samples years before diagnosis, clinical translation is limited by both low sensitivity and reduced specificity due to age-related and tissue-specific clonal expansions. These limitations have driven a shift toward broader genomic approaches that capture global hallmarks of malignancy, particularly chromosomal instability and copy number alterations, as complementary or alternative detection strategies.

### 3.4. Beyond Single Genes: Genomic and Multi-Gene Panel Strategies

The inherent challenges of detecting a single, low-frequency point mutation (such as TP53) with sufficient sensitivity and specificity drove the development of complementary molecular strategies. These approaches aim to cast a wider net, either by capturing broader genomic signatures of malignancy or by simultaneously screening for a panel of driver mutations. Given the sensitivity and specificity limitations of single-gene mutation analysis, investigators have increasingly explored genome-wide alterations as more robust biomarkers.

#### 3.4.1. Detection of Genomic Instability and Copy Number Alterations

Building on this rationale, analysis has expanded from single-gene mutations to broader signatures of chromosomal instability. Paracchini et al. developed a test (EVA—early Ovarian cancer test) based on the analysis of somatic copy number alterations (SCNAs) to address the sensitivity challenges of mutation-based detection [29]. In a retrospective, multicentric study analyzing 250 archival Pap test samples from women later diagnosed with HGSOC and matched controls, the team employed low-pass whole-genome sequencing. They quantified genomic instability using a copy number profile abnormality (CPA) score, a composite metric derived from genome-wide copy number segmentation that summarizes the extent and amplitude of chromosomal gains and losses across the genome. Samples from women who later developed HGSOC exhibited significantly higher CPA values, reflecting an increased burden of SCNAs detectable up to nine years prior to diagnosis. The EVA test, integrating the CPA score, demonstrated a sensitivity of 75% (95% CI, 64.97–85.79) and a specificity of 96% (95% CI, 88.35–100.00) in distinguishing these pre-diagnostic samples from healthy controls. However, these performance estimates derive from retrospective analysis of archival samples with defined pre-analytical conditions and have not yet been validated in prospective screening settings, where test performance may differ.

Taken together, these findings suggest that assessment of chromosomal instability in Pap smear DNA may have potential for early detection of HGSOC, although this remains to be confirmed in prospective studies.

#### 3.4.2. Somatic Mutation Detection with Multi-Gene Panels

In parallel, to improve sensitivity and scalability, significant efforts have focused on detecting a spectrum of somatic mutations via multi-gene panels, without reliance on prior tumor sequencing. Wang et al. developed PapSEEK, a multiplex PCR-based test for mutations in 18 driver genes combined with aneuploidy analysis in DNA from cervicovaginal fluid [5]. In a retrospective study of 245 OC patients, the assay’s sensitivity using standard Pap brush samples was 33% (95% CI, 27–39%) with a specificity of 99%. Using a novel intrauterine Tao brush in a subset of 51 patients increased sensitivity to 45% (95% CI, 31–60%) at 100% specificity (0/125 controls), highlighting the significant impact of sampling method; however, the Tao brush is not part of standard cervical cancer screening practice, which may limit the generalizability of these findings. For 83 patients with paired samples, combining Pap brush analysis with plasma circulating tumor DNA (ctDNA) testing increased overall detection to 63% (95% CI, 51–73%), underscoring the potential of a multi-analyte “liquid biopsy” approach. However, the routine implementation of combined cervicovaginal and plasma ctDNA testing in a population screening setting has not been evaluated in prospective screening trials, and its feasibility in such a context remains to be established and is currently uncertain.

Other ultrasensitive platforms and focused gene panels have been explored to maximize detection from limited DNA. In a pilot observational study, Jiang et al. assessed the feasibility of detecting tumor-derived somatic mutations in Pap smear DNA and plasma cell-free DNA (cfDNA) using the cSMART assay, an ultrasensitive method with a detection limit of 0.01% mutant allele frequency [30]. After constructing an 8-gene OC-specific panel from tumor whole-exome sequencing, they analyzed matched Pap smear DNA from 11 patients. Tumor-associated signals were detected in 100% (11/11) of Pap samples. However, among 10 cases with matched tumor sequencing data, identical somatic mutations were found in only 5 cases (50%), indicating partial concordance and suggesting the presence of non-tumor-derived or subclonal variants in the cervical sample. These non-concordant signals may reflect either technical false positives inherent to ultra-sensitive assays or, more plausibly, biologically distinct somatic clones arising from background epithelial or age-related clonal processes, rather than true tumor-derived mutations. In contrast, plasma cfDNA demonstrated superior concordance with the primary tumor (71.4%, 10/14 evaluable cases), reinforcing that plasma may more faithfully reflect the dominant tumor clone and supporting the evaluation of combined cervicovaginal and blood-based testing.

In a prospective multicenter study, van Bommel et al. performed targeted NGS on cervicovaginal self-samples, Pap smears, and pipelle endometrial biopsies from 29 OC patients and 32 controls [31]. While pathogenic variants were identified in 83% of surgical tumor specimens, the detection rate in pre-operative samples was lower. A variant was found in 52% of patients in at least one cervicovaginal or endometrial sample. The Pap smear yielded the highest individual sensitivity at 26% (95% CI, 10–48%). The study concluded that while specificity was high (>94%), the sensitivity of mutation detection in these samples was currently insufficient for reliable early detection.

Focusing on tumor characteristics and biospecimen source, Kubo-Kaneda et al. performed NGS on cervical and endometrial LBC samples from 19 OC patients [32]. Pathogenic variants (TP53, PIK3CA, RB1) concordant with the matched tumor tissue were detected in 42% of cases overall, with a higher rate of 54.5% for HGSOC. In univariate analysis of HGSOC cases, the presence of a serous tubal intraepithelial carcinoma (STIC) showed a suggestive but not statistically significant association with successful detection in LBC samples (HR 20.0, 95% CI 0.93–429.9; *p* = 0.056), with the wide confidence interval reflecting the very small sample size.

Thus, while multi-gene panels and SCNA analysis represent significant advances, biological barriers to sensitivity persist, motivating the exploration of entirely different molecular target classes.

### 3.5. Emerging Multi-Omic Frontiers

The exploration of Pap test biospecimens has extended beyond the genome into the epigenome and proteome. These “multi-omic” approaches aim to uncover alternative, stable molecular signatures of OC that may offer advantages in sensitivity, specificity, or detection of early biological changes.

#### 3.5.1. DNA Methylation Biomarkers in Cervical Smears

Beyond mutational analysis, the detection of tumor-specific DNA methylation patterns in cervical smears presents a highly promising molecular strategy for OC detection. In a seminal study, Wu et al. integrated methylomic profiles from ovarian tissues and cervical smears to identify a panel of three hypermethylated genes—AMPD3, NRN1, and TBX15 [33]. This targeted panel, validated by quantitative methylation-specific PCR, demonstrated a sensitivity of 81% and a specificity of 84% for detecting OC in a testing set of cervical scrapings, achieving an area under the curve (AUC) of 0.91 (95% CI, 0.82–1). These estimates were derived from a relatively small, case–control testing set of cervical scrapings, which may limit generalisability to broader screening populations. The study also noted that the performance differed by histology, with mucinous carcinomas exhibiting a significantly lower methylation risk score than non-mucinous subtypes, suggesting lineage-specific epigenetic profiles that may have implications for subtype-specific sensitivity in a screening context. Building upon this concept of methylation-based detection, Lien et al. employed a machine-learning approach on genome-wide methylation data from cervical smears to develop a clinically nuanced, two-step stratification model [34]. Their model first distinguished normal from tumor samples (benign and malignant combined) using a 30-feature principal component analysis model (AUPRC 0.95, F1-score 0.93 in training). A subsequent gradient boosting model, utilizing 16 CpG sites, then classified the tumor samples as benign or malignant (AUPRC 0.98, F1-score 0.92). The use of AUPRC (area under the precision–recall curve), rather than the more commonly reported AUC-ROC, is particularly appropriate in this context, as it provides a more informative assessment of model performance in class-imbalanced datasets by focusing on precision and recall of the minority (tumor) class, whereas AUC-ROC may overestimate performance when the negative class predominates. On an independent testing set, the combined model correctly stratified 35 of 40 samples (88% accuracy) with an overall F1-score of 0.86, effectively differentiating not only cancer from normal but also addressing the critical diagnostic challenge of discriminating benign from malignant adnexal pathology.

O’Keefe et al. developed a novel microfluidic platform, PapDREAM, for single-molecule digital high-resolution melt analysis of DNA methylation heterogeneity [35]. They applied this platform to a custom panel of nine methylated loci in DNA from Pap specimens of 18 OC patients and 25 healthy controls. Their test achieved a clinical sensitivity of 50% at a specificity of 99%, with an area under the curve (AUC) of 0.90. A simplified three-gene model maintained an AUC of 0.88. Their analysis revealed that optimal detection for biomarkers like ZNF154 occurred at an intermediate 40% methylation density threshold, highlighting the platform’s ability to capitalize on heterogeneous epigenetic alterations often present in early disease.

Collectively, this work establishes DNA methylation in cervical samples as a detectable target, achievable via targeted, agnostic, and single-molecule methods with clinically relevant accuracy.

#### 3.5.2. Proteomic Profiling for Biomarker Discovery

Beyond nucleic acids, mass spectrometry (MS)-based proteomics offers an untargeted approach for biomarker discovery. Boylan et al. conducted a comprehensive proteomic comparison of matched biospecimens from a patient with HGSC: primary tumor tissue, a cervical swab, and cell-free Pap test fluid [36]. Using 2D-LC MS/MS, they identified a total of nearly 5000 proteins, with a core set of 2293 proteins common to all three biospecimens. Established OC serum biomarkers, including CA125 (MUC16), HE4 (WFDC2), and mesothelin, were identified in both the Pap test fluid and the cervical swab. Other proteins elevated in OC, such as leucine-rich alpha-2-glycoprotein (LRG) and CD44, were also detected. This proof-of-concept study confirms that Pap test fluid and cervical swabs harbor a rich source of tumor-associated proteins, establishing a foundation for the future development of targeted, quantitative protein assays.

### 3.6. Case Reports—The Incidental Detection of Extra-Cervical Gynecological Malignancies

The clinical implications of cytological and molecular findings are illustrated by case reports describing incidental detection of extra-cervical malignancies on Pap smears. Across these reports, malignant glandular cells or AGC-type findings frequently represented the first diagnostic clue to tumors originating from the fallopian tube, peritoneum, and ovary, often in asymptomatic patients. Primary fallopian tube and peritoneal carcinomas were typically identified through adenocarcinoma or atypical glandular cytology on LBC, sometimes showing papillary or “berry-like” clusters, and consistently required further adnexal and peritoneal evaluation following negative cervical and endometrial work-up [37,38,39,40,41].

Ovarian neoplasms were also represented, including serous borderline tumors, HGSOC with associated STIC, and rare malignant transformations in dermoid cysts, highlighting the broad pathological spectrum that may underlie abnormal cervical cytology [42,43,44].

Additional studies demonstrate the added diagnostic value of residual LBC material, which can be processed into cytoblocks for immunohistochemical and phenotypic refinement (e.g., CK7/CK20, p16), improving tumor classification and aiding in the determination of primary origin [45]. Importantly, not all AGC findings reflect epithelial malignancy, as illustrated by rare mesenchymal lesions such as UTROSCT, underscoring potential diagnostic pitfalls [46].

Collectively, these case reports highlight that abnormal cervicovaginal cytology may represent the first indication of clinically occult extra-uterine malignancy, but also that accurate interpretation requires integration with imaging and ancillary molecular or immunohistochemical techniques to establish definitive diagnosis.

## 4. Synthesis and Future Directions

In the context of secondary prevention, existing strategies such as CA-125-based algorithms and TVUS have not demonstrated a clear mortality benefit in large trials, despite improving detection rates. In contrast, Pap-based molecular approaches represent a fundamentally different strategy, leveraging cervicovaginal biospecimens to detect tumor-derived signals originating from the upper genital tract. While this review focuses on Pap smear–derived material, alternative sampling approaches such as tampon-based collection, menstrual blood, or uterine lavage have also been explored, although these are not yet standardized or integrated into routine screening practice. While promising, these approaches remain investigational and currently lack prospective evidence demonstrating superiority over, or complementarity with, established screening modalities.

Against this backdrop, the evidence reviewed demonstrates that while the Pap test can capture both cellular and molecular evidence of OC (Table 1), its clinical application is constrained by a fundamental biological limit: the scant and sporadic shedding of tumor material into the cervical canal. This reality dictates that a Pap-based test for OC will not function as a conventional, high-sensitivity screening tool for the general population. Instead, the most viable and promising pathway forward is to integrate molecular Pap testing into a defined clinical algorithm as a powerful risk-stratification step.

**Figure 2:**
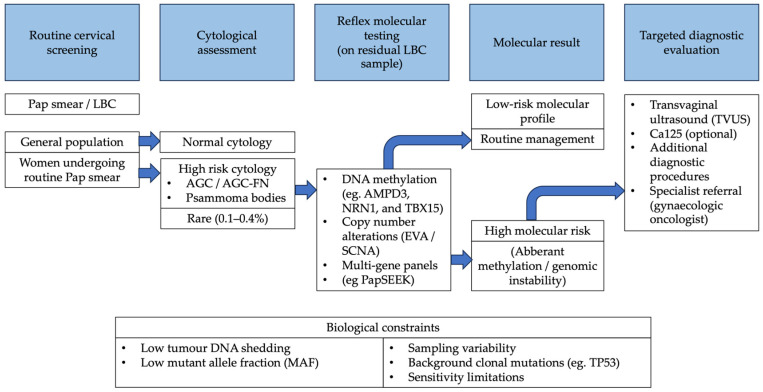
Reflex-to-Molecular workflow for OC risk stratification using Pap smear samples.

Collectively, the evidence points toward a practical, integrated clinical model. The routine Pap smear would serve as a universal biospecimen collection. For the vast majority with normal cytology, no further action is taken. However, for the small subset of women with specific, high-risk cytological findings (such as AGC, particularly AGC-FN, or the incidental finding of PB), the residual LBC fluid would automatically undergo reflexive molecular testing. This second-tier analysis could employ the most robust signatures identified, such as a targeted DNA methylation panel (e.g., AMPD3/NRN1/TBX15) or a genomic instability score (EVA test), to objectively quantify the risk of an underlying OC. A high-risk molecular result would then mandate prompt and focused diagnostic investigation with imaging and specialist referral. This “reflex-to-molecular” model efficiently leverages the existing screening infrastructure, targets resources to the highest-risk patients, and provides a concrete diagnostic pathway for managing ambiguous cytology (Figure 2).

Although these molecular assays (including ddPCR, targeted NGS, and methylation-based panels) are analytically robust, their current cost and laboratory requirements may limit widespread implementation in population-level screening programs. Importantly, formal health-economic evaluations of cervicovaginal molecular testing strategies are still lacking. In this context, a reflex-to-molecular approach, restricted to cytologically or clinically high-risk subgroups, may represent a more feasible and cost-efficient translational pathway than universal molecular screening.

It is important to emphasize that most molecular evidence reviewed in this manuscript is derived from studies of HGSOC, which reflects both its epidemiological predominance and its distinct pathogenesis originating from the fallopian tube epithelium. Other histological subtypes of OC, including clear cell, endometrioid, mucinous, and low-grade serous carcinomas, exhibit fundamentally different molecular landscapes and are therefore unlikely to be captured with the same sensitivity by TP53-centered mutation assays, SCNA-based approaches, or current methylation signatures. As such, the clinical performance of the proposed reflex-to-molecular model across non-serous OC subtypes remains uncertain and will require dedicated subtype-specific validation in future studies.

An additional consideration for clinical translation is the substantial heterogeneity in pre-analytical conditions across the included studies, which may influence the performance of molecular assays in cervicovaginal samples. Differences in LBC platforms (e.g., ThinPrep vs. SurePath), sample collection devices, storage time and conditions, DNA extraction protocols, and, for methylation-based assays, bisulfite conversion efficiency, are likely to affect assay sensitivity and reproducibility. This is particularly relevant for low-abundance analytes such as tumor-derived DNA, where small variations in processing can meaningfully impact detection rates. As most studies did not systematically standardize or report these parameters, cross-study comparisons should be interpreted cautiously. Future clinical implementation will require harmonization of pre-analytical workflows and standardized reporting of sample processing to ensure reproducibility and comparability across settings.

To realize this model, focused translational research is required. The immediate priority is prospective clinical validation of a combined cytology–molecular reflex protocol specifically in women with high-risk cytological findings, such as AGC (particularly AGC-FN), comparing its diagnostic accuracy, downstream clinical management, and outcomes to current standard care. Concurrently, technical standardization of pre-analytical (sample collection, storage, LBC platform) and analytical (assay selection and thresholds) workflows is essential to ensure reproducibility across clinical settings. Finally, formal cost-effectiveness analyses are needed to define the optimal clinical use case, which is unlikely to be population-wide screening, but rather targeted application in high-risk cytology cohorts or in the surveillance of genetically predisposed individuals (e.g., BRCA carriers).

An additional consideration for clinical implementation is the ethical dimension of extending molecular analyses to Pap smear material originally collected for cervical cancer screening. This includes issues of informed consent for secondary genomic testing, the management of incidental findings such as age-related somatic mutations or clonal hematopoiesis, and the potential psychological impact of false-positive results in asymptomatic individuals. These challenges highlight the need for clear consent frameworks, standardized reporting, and defined clinical pathways prior to routine use.

Despite these promising findings, several limitations should be acknowledged. As a narrative review, it lacks the methodological rigor of a systematic review, including a formal quality assessment of included studies. The evidence base is heterogeneous, comprising case reports, small observational studies, and predominantly retrospective analyses, which introduces potential selection bias and limits the generalizability of findings. Moreover, the absence of prospective validation studies precludes conclusions regarding clinical utility, including impact on survival or mortality reduction. Restriction to published studies also raises the possibility of publication bias, with underrepresentation of negative findings. Finally, cost-effectiveness analyses of Pap-based molecular strategies are currently lacking and will be essential for evaluating their feasibility in clinical practice.

## 5. Conclusions

In summary, the future of Pap test-based OC detection lies not in reinventing it as a standalone screen, but in strategically augmenting it. By using advanced molecular analysis to clarify the significance of rare but concerning cytological findings, this familiar test could be transformed into a sophisticated triage tool, guiding the timely diagnosis of OC in high-risk groups, such as postmenopausal women with AGC (particularly AGC-FN) or PB, and genetically predisposed individuals (e.g., BRCA carriers) with inconclusive or negative imaging.

## Figures and Tables

**Table 1:** Summary of Investigated Cytological and Molecular Biomarkers in Pap Smear for OC Detection.

Biomarker/Group	Study Design	Sample Size	Mechanism/Role	Association with OC Detection	Validation Status
Atypical Glandular Cells (AGC)	Retrospective cohort studies	Large population-based cohorts (registry-level; variable n)	Cytologic marker of glandular atypia; possible transtubal shedding	SIR 2.04; OC in 0.6–2.5% of AGC cases; higher in AGC-FN	Epidemiologically validated (multiple population cohorts)
Psammoma Bodies (PB)	Retrospective case series + case reports	Very large screening cohorts (prevalence studies up to >1.4 M smears) + small outcome series	Calcified structures linked to serous tumors	Strong association with serous OC; 87.5% malignancy in postmenopausal cases (small series)	Limited clinical evidence (rare-event observational data)
TP53 mutations	Retrospective case–control + longitudinal archival studies	Small cohorts (~15–30 OC patients per study)	Detection of tumor-derived point mutations in cervicovaginal DNA	Detectable up to 6 years pre-diagnosis; sensitivity ~30–75%	Analytically validated (retrospective); no prospective validation
Somatic Copy Number Alterations (SCNAs)	Retrospective	~250 archival Pap samples	Genome-wide chromosomal instability (CPA score)	Sensitivity 75%, specificity 96%; detectable up to 9 years pre-diagnosis	Retrospective validation only; no prospective validation
Multi-gene mutation panels ± aneuploidy (e.g., PapSEEK)	Retrospective case–control	Moderate case–control cohorts (e.g., ~245 OC patients + controls)	Detection of mutations across multiple driver genes with aneuploidy assessment	Sensitivity 33–45%, specificity up to 100% (sampling-dependent; higher with intrauterine sampling)	Retrospective validation only; no prospective validation
DNA methylation panels (e.g., AMPD3, NRN1, TBX15)	Case–control biomarker discovery studies	Small–moderate training/testing cohorts	Detection of tumor-specific DNA hypermethylation signatures	Sensitivity up to 81%, specificity 84%	Retrospective validation only; no prospective validation
Single-molecule methylation analysis (PapDREAM)	Pilot case–control	Small pilot cohort (e.g., 18 OC + 25 controls)	Digital profiling of methylation heterogeneity at single-molecule level	Sensitivity 50%, specificity 99%, AUC 0.90	Preliminary evidence (pilot study; no external validation)
Liquid-based cytology + IHC (CK7, p16, etc.)	Retrospective diagnostic studies + case series	Small heterogeneous cohorts	Protein expression for tumor origin classification	Improves diagnostic specificity of cytologic interpretation (not a primary detection method)	Clinically established diagnostic adjunct; not validated for screening
Proteomic profiles (CA125, HE4, LRG, etc.)	Proof-of-concept proteomic study	Exploratory datasets (single-patient and small cohorts)	Detection of tumor-associated proteins in Pap fluid and cervical samples	Demonstrates presence of known OC biomarkers in cervicovaginal samples	Discovery stage (no validation cohorts)

## Data Availability

No new data were created or analyzed in this study.

## Supplementary Materials

The following supporting information can be downloaded at: https://www.mdpi.com/article/10.3390/medicina62050873/s1, Figure S1: Prisma diagram. Reference [47] is cited in the supplementary materials.

## Abbreviations

The following abbreviations are used in this manuscript:

Pap smear	Papanicolaou smear
AGC	Atypical glandular cells
AGC-FN	AGC-favor neoplastic
PB	Psammoma bodies
OC	Ovarian cancer
HGSOC	High-grade serous ovarian carcinoma
IHC	Immunohistochemistry
HPV	Human papillomavirus
DNA	Deoxyribonucleic acid
cfDNA	Plasma cell-free DNA
ctDNA	Plasma circulating tumor DNA
TP53	Tumor protein P53
