# Supplementary material for: The Papanicolaou Smear Reimagined: A Narrative Review of Cervicovaginal Cytology and Molecular Biospecimens for Ovarian Cancer Detection
Source: Medicina (Kaunas). 2026 May 2;62(5):873. doi: 10.3390/medicina62050873 (PMC13208270; doi:10.3390/medicina62050873)
Supplement: Supplementary file 1 [file medicina-62-00873-s001.zip › medicina-4235107-supplementary.pdf]

PRISMA 2020 flow diagram for new systematic reviews which included searches of databases and registers only

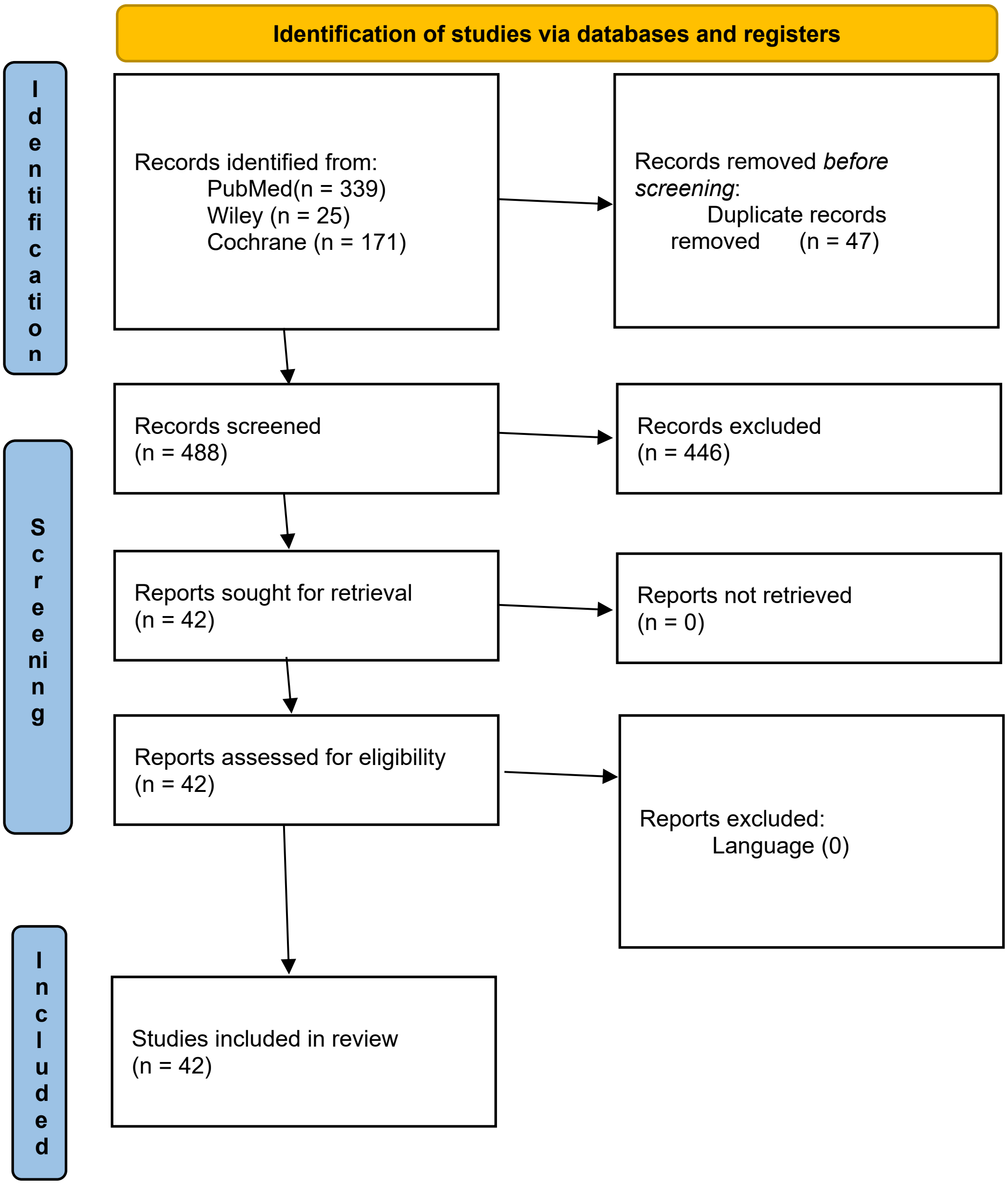

*Consider, if feasible to do so, reporting the number of records identified from each database or register searched (rather than the total number across all databases/registers).

**If automation tools were used, indicate how many records were excluded by a human and how many were excluded by automation tools.

Source: Page MJ, et al. BMJ 2021;372:n71. doi: 10.1136/bmj.n71.

This work is licensed under CC BY 4.0. To view a copy of this license, visit https://creativecommons.org/licenses/by/4.0/
